# Assessment of the Preventive Effect Against Diabetic Cardiomyopathy of FGF1-Loaded Nanoliposomes Combined With Microbubble Cavitation by Ultrasound

**DOI:** 10.3389/fphar.2019.01535

**Published:** 2020-01-10

**Authors:** Lei Zheng, Chuan-Li Shen, Jian-Min Li, Yu-Lei Ma, Ning Yan, Xin-Qiao Tian, Ying-Zheng Zhao

**Affiliations:** ^1^ Department of Ultrasonography, Henan Provincial People’s Hospital, Zhengzhou University People’s Hospital, Department of Ultrasonography of Central China Fuwai Hospital, Central China Fuwai Hospital of Zhengzhou University, Zhengzhou, China; ^2^ Department of Ultrasonography, the First Affiliated Hospital of Wenzhou Medical University, Wenzhou, China; ^3^ Department of Pathology, the First Affiliated Hospital of Wenzhou Medical University, Wenzhou, China; ^4^ School of Pharmaceutical Sciences, Wenzhou Medical University, Wenzhou, China; ^5^ Engineering Laboratory of Zhejiang Province for Pharmaceutical Development of 6 Growth Factors, Biomedical Collaborative Innovation Center of Wenzhou, Wenzhou, China

**Keywords:** diabetic cardiomyopathy, acidic fibroblast growth factor, ultrasound-targeted microbubble destruction, liposomes, preventive effect

## Abstract

Acidic fibroblast growth factor (FGF1) has great potential in preventing diabetic cardiomyopathy. This study aimed to evaluate the preventive effect of FGF1-loaded nanoliposomes (FGF1-nlip) combined with ultrasound-targeted microbubble destruction (UTMD) on diabetic cardiomyopathy (DCM) using ultrasound examination. Nanoliposomes encapsulating FGF1 were prepared by reverse phase evaporation. DM model rats were established by intraperitoneal injection of streptozotocin (STZ), and different forms of FGF1 (FGF1 solution, FGF1-nlip, and FGF1-nlip+UTMD) were used for a 12-week intervention. According to the transthoracic echocardiography and velocity vector imaging (VVI) indexes, the LVEF, LVFS, and VVI indexes (Vs, Sr, SRr) in the FGF1-nlip+UTMD group were significantly higher than those in the DM model group and other FGF1 intervention groups. From the real-time myocardial contrast echocardiography (RT-MCE) indexes, the FGF1-nlip+UTMD group A and A×β showed signiﬁcant differences from the DM model group and other FGF1 intervention groups. Cardiac catheter hemodynamic testing, CD31 immunohistochemical staining, and electron microscopy also confirmed the same conclusion. These results confirmed that the abnormalities, including myocardial dysfunction and perfusion impairment, could be suppressed to different extents by the twice weekly FGF1 treatments for 12 consecutive weeks (free FGF1, FGF1-nlip, and FGF1-nlip+UTMD), with the strongest improvements observed in the FGF1-nlip+UTMD group. In conclusion, the VVI and RT-MCE techniques can detect left ventricular systolic function and perfusion changes in DM rats, providing a more effective experimental basis for the early detection and treatment evaluation of DCM, which is of great significance for the prevention of DCM.

## Introduction

With the development of the economy and society, as well as changes in people’s dietary habits and lifestyles, the incidence of diabetes mellitus (DM) is increasing annually, and the age of onset has become younger. According to statistics, there are approximately 425 million DM patients worldwide in 2017, and the number is expected to reach 700 million by 2045 (Cho et al., 2018). The acute and chronic complications caused by DM not only endanger the physical and mental health of patients but also bring a huge economic burden to patients’ families and the health industry. Diabetes cardiomyopathy (DCM) is a series of changes in the myocardial structure and function caused by DM (Borghetti et al., 2018; Parim et al., 2019) that are not related to coronary atherosclerosis, hypertension, and valvular heart disease (Rubler et al., 1972). At present, DCM has been identified as the main cause of heart failure and death in patients with DM (Evangelista et al., 2019; Gulsin et al., 2019; Zamora and Villena, 2019). Therefore, early detection and timely intervention therapy are of great significance for the prognosis of DM patients.

Fibroblast growth factors (FGFs) have at least 23 members and are widely distributed in the body. Each member has a certain structural similarity (Beenken and Mohammadi, 2009; Itoh and Ornitz, 2011; Zhang et al., 2013; Li, 2019). Among them, acidic fibroblast growth factor (FGF1) is one of the earlier founded members of the fibroblast growth factor family. Studies have shown that FGF1 has a wide range of physiological functions, such as antioxidant damage (Pena et al., 2017), induction of endothelial and smooth muscle cell proliferation and angiogenesis (Zhao et al., 2016), promotion of tissue wound repair (Xu et al., 2018), neurotrophic regeneration (Tsai et al., 2015), etc. Therefore, FGF1 has a great potential in the prevention and treatment of DCM. However, the lack of an efficient and safe delivery system limits FGF1 application *in vivo*.

Nanoliposomes are double-layer vesicle structures composed of hydrated lipids (Abu and Ishida, 2017; Kapoor et al., 2017). The particle sizes range from 10 to 1000 nm. Nanoliposomes have the characteristics of high drug loading, sustained-release drugs, high stability *in vivo*, non-toxicity, non-immunogenicity, and biodegradability (Chen and Stephen, 2019). Therefore, using nanoliposomes as drug carriers can not only effectively protect the drug from the influence of environmental conditions *in vivo* but also can penetrate the endothelial gap and complete capillaries to reach the target tissue to be absorbed by most cells, thus playing a corresponding biological effect. However, it is difficult for nanoliposomes to locally aggregate at high concentrations *in vivo* to achieve highly effective targeted therapy.

Ultrasound targeted microbubble destruction (UTMD) provides a new method for myocardial targeted delivery of FGF1. Under the energy of diagnostic or therapeutic ultrasound, ultrasound microbubbles can explode in the region of interest or target tissues. Cavitation and mechanical effects of blasting can increase the permeability of local vascular walls or cell membranes, thereby increasing the dose of drugs/genes in target organs or target tissues and their corresponding biological effects (Frenkel, 2008; Chen et al., 2018; Liang et al., 2018; Lin et al., 2018; Yang et al., 2019).

Echocardiography, as a practical tool for the non-invasive evaluation of cardiac function, has been widely applied in clinical and animal experiments. Velocity vector imaging (VVI) is based on two-dimensional gray-scale ultrasound images with a high frame rate. It uses spatial coherence, speckle and boundary tracking techniques of ultrasound pixels to automatically track and recognize the motion of echo spots in the region of interest in each frame image and quantitatively analyzes the structural mechanics of myocardial tissue motion to obtain a reflection of the myocardium. Compared with traditional techniques, VVI has no angle dependence, and its strain and strain rate measurements are relatively unaffected by respiration (Azam et al., 2012; Li et al., 2012; Zhou et al., 2015). Therefore, it is superior to conventional ultrasound and tissue imaging and its derivative technology in cardiac function abnormalities and heart disease treatment effect evaluation. Real-time myocardial contrast echocardiography (RT-MCE) technology is used to inject ultrasound contrast agents containing microbubbles into the body through peripheral veins. Because the size of microbubbles is the same as that of red blood cells, the hemodynamics is similar to that of red blood cells. Microbubbles can freely distribute in myocardial tissue through cardiac capillaries. Microbubbles produce a large number of liquid-gas interfaces in the blood, thus reflecting a large number of ultrasound signals and increasing the video density of myocardial microcirculation. By observing the contrast enhancement of the myocardium with echocardiography, the tissue perfusion information can be evaluated at the microvascular level. The whole and local perfusion of myocardium can be observed and analyzed non-invasively. The volume, velocity, and flow of myocardium can be measured quantitatively (Wei et al., 2012; Jiang et al., 2017; Danijela et al., 2018; Geng et al., 2018). Although the application of VVI and RT-MCE technology is increasing, few studies have evaluated the efficacy of DCM in prevention and treatment.

Therefore, FGF1 loaded nanoliposomes (FGF1-nlip) combined with UTMD technology were used in this study to intervene in early DM rats. The effects of this method on left ventricular function and blood flow perfusion in DM rats were evaluated by conventional echocardiography, velocity vector imaging (VVI), real-time myocardial contrast echocardiography (RT-MCE), and histomorphology.

## Materials and Methods

### Preparation and Properties of FGF1-nlip

FGF1-nlip were prepared by reverse phase evaporation. The specific preparation process was as follows: FGF1 lyophilized powder (Guangzhou Jinan University Medical Biotechnology Research and Development Center) was dissolved in physiological saline to form FGF1 (1 mg/ml) solution. Next, 90 mg of natural phospholipids (Shanghai AVT) and 10 mg of cholesterol (Shanghai AVT) were weighed and dissolved in 4 ml of dichloromethane (Guangdong Guanghua Technology Co., Ltd.) for use. The FGF1 solution was slowly added dropwise to the phospholipid-dissolved methylene chloride and rapidly sonicated (100 W, 15 s) to form a stable w/o emulsion. The methylene chloride was removed by rotary evaporation under reduced pressure. After the dichloromethane was completely removed, the mixture was hydrated with an appropriate amount of physiological saline and ultrasonically dispersed (100 W, 10 min) in an ice bath to give a final FGF1 concentration of 10 μg/ml. Blank nanoliposomes were prepared by replacing the FGF1 solution with an equal volume of distilled water. The characterization of FGF1-nlip mainly includes morphological characteristics, zeta potential, particle size, and the encapsulation efficiency of the particles. In this study, the particle size and morphological characteristics of FGF1-nlip were determined by transmission electron microscopy. The hydration particle size and zeta potential of FGF1-nlip were determined by dynamic light scattering. The determination method of the encapsulation efficiency of FGF1-nlip was as follows: 1.0 ml of liposome suspension containing FGF1 was accurately measured in an ultrafiltration tube, centrifuged at 10,000 r/min for 30 min, the filtrate was removed and diluted properly, and the FGF1 ELISA reagent was used. The cassette was assayed for FGF1 protein concentration. Encapsulation ratio (%) = (total protein amount − amount of protein in the filtrate)/total protein amount × 100%.

### Experimental Animals

Seventy male Sprague-Dawley (SD) rats, aged 40 to 50 days, with body weight 180–220 g, were provided by the Experimental Animal Center of Wenzhou Medical University (Experimental Animal License No.: SYXK (Zhejiang] 2015-0009). All animal experiments were performed with the permission of the Animal Ethics Committee of Wenzhou Medical University (approval number: wydw2016-0025).

### Experimental Animal Groups

After 7 days of adaptive feeding, all rats were fasted for 12 hours overnight. On the second day, 56 rats were randomly selected, and each rat was intraperitoneally injected with a 1% solution of streptozotocin 70 mg/kg. The blood glucose concentration was measured by tail vein sampling on the 1st, 3rd, and 7th days after injection. The rats with blood glucose levels exceeding 16.7 mmol/L and with higher food intake, polydipsia, and polyuria were selected as DM rats (52 rats were modeled, 4 were excluded without being modeled). Subsequently, DM rats were randomized into 4 groups with 13 rats in each group: DM model group, FGF1 solution group, FGF1-nlip group, and FGF1-nlip+UTMD group. The remaining 14 were used as the normal control group, and the same amount of citrate buffer was intraperitoneally injected and they were fed under the same conditions.

All experimental rats were housed in the SPF animal room (the average indoor temperature was approximately 20–24°C, the average humidity was approximately 50–60%), and the on-time changes were simulated by turning on and off the lights regularly. All rats were freely supplied with sterile water during the feeding process.

### Experimental Animal Intervention

After intraperitoneal anesthesia with 10% chloral hydrate solution, rats in the DM model group were injected with saline *via* the caudal vein; rats in the FGF1 solution group were injected with FGF1 solution *via* the caudal vein; and rats in the FGF1-nlip group were injected with FGF1-nlip solution *via* the caudal vein. After anesthesia, rats in the FGF1-nlip+UTMD group were shaved in the anterior cardiac region and fixed in the left lateral decubitus position. FGF1-nlip solution was well mixed with SonoVue microbubbles (Bracco, Italy) before administration. The Acuson Sequoia 512C system (Siemens, Germany) was used, and the RT-MCE imaging mode was set up. The probe frequency was set to 12 to 14 MHz (15L8-w linear array probe). The probe was placed in the precordial area of the rats, and the probe and skin were filled with coupling agents. The ultrasound instrument was converted into contrast mode. Left ventricular short axis view at the papillary muscle level was taken, and the focusing depth was 3.5–4.0 cm. The mixture of FGF1-nlip and SonoVue microbubbles was slowly injected into rats. When the image showed that a large number of contrast agents were filling the myocardium, MBD function (mechanical index MI = 1.9) was used to blast the microbubbles repeatedly until the microbubbles completely disappeared. The dose of fibroblast growth factor 1 in each intervention group was 15 µg/kg (Zhao et al., 2016). Drug intervention lasted 12 weeks, twice a week, for a total of 24 times.

### Ultrasound Image Acquisition

Before and 12 weeks after the intervention (De Blasio et al., 2015; Atta et al., 2018), rats in each group were anesthetized with 10% chloral hydrate intraperitoneally and shaved in the anterior cardiac region. The probe was placed in the anterior cardiac region of rats, and the coupling agent was filled between the probe and the skin. The left ventricular ejection fraction (LVEF) and left ventricular fraction shortening (LVFS) were measured by conventional M-mode echocardiography in all rats.

VVI imaging mode was started, and the RES button on the machine was pressed to enlarge the image while reducing the spatial resolution appropriately to obtain the highest frame rate possible. The frame rate of this study was adjusted to between 60 and 90 Hz. Acoustic acquisition of two-dimensional gray-scale dynamic images of the papillary muscle horizontal left ventricular short axis view for three or more cardiac cycles was used. All dynamic images were stored on MO discs and imported into Siemens Sygno US Workplace 3.01 analysis software for offline analysis.

The cadence key was pressed to enter the contrast mode. The depth of the image was 3–4 cm. The RES key was pressed to enlarge the region of interest locally. The mechanical index was 0.35. The gain was adjusted so that there was no obvious acoustic signal in the myocardial tissue and remained constant throughout the process. Contrast medium was infused slowly through the caudal vein. Left ventricular short axis myocardiography at the papillary muscle level was performed at rest to observe the filling of contrast media in the cardiac cavity and myocardium. After the filling of contrast media in the myocardium was sufficient, the contrast media in the myocardium were destroyed by high energy pulse, and then the instrument was automatically converted to low energy imaging to obtain real-time dynamic imaging. All real-time dynamic images were stored on MO discs and then imported into Siemens Sygno US Workplace 3.01 analysis software for off-line auto-tracing contrast quantitative technique (ACQ) analysis.

### VVI Analysis

In the VVI analysis software, a frame image of the clearest endocardium was selected, and the boundary of the left ventricular endocardium and epicardium was drawn clockwise. According to the standard 16-segment method developed by the American Academy of Echocardiography, the left ventricular wall was automatically divided into six segments: anterior wall (AW), lateral wall (LW), posterior wall (PW), inferior wall (IW), posterior septum (PS), and anterior septum (AS). The position of each segment was monitored, and the myocardial velocity, radial strain, and strain rate curves of 6 segments were obtained automatically by software. The mean systolic peak velocity (Vs), radial peak strain (Sr), and radial peak strain rate (SRr) were measured in 6 segments of the left ventricle.

### MCE Analysis

In the analysis software, the left ventricular short-axis view of the anterior wall myocardial tissue was selected to outline the region of interest (in the process of delineation, the interference of factors such as endometrial, epicardial, and papillary muscles was avoided). The software automatically tracks the region of interest in each frame of the image and allows the operator to adjust the location of the region of interest at any frame so that it is always within the corresponding myocardial tissue. The analysis software measures the average acoustic intensity of the region of interest of each frame of image and fits it to the acoustic intensity-time curve y = A × (1 − e^-βt^), where A is the peak acoustic intensity of the region of interest, reflecting the blood volume of the local myocardium, β is the contrast agent perfusion rate, reflecting the blood flow velocity of the myocardium, the product of the two (A×β) represents the regional myocardial blood flow, and each rat is analyzed 2~3 times and the average taken to reduce the error. All of the analysis curves with a goodness of fit (GOF) < 0.9 were excluded.

### Hemodynamic Evaluation

All the experimental rats were anesthetized with 10% chloral hydrate after intraperitoneal injection, and then they were routinely disinfected. The skin of the anterior cervical region was cut, the trachea was separated, the animal ventilator was opened, the thoracic cavity was opened, and the cardiac catheter was penetrated into the left ventricle of the rat with heparin saline after filling the heart catheter with heparin saline. After the biological signal was transformed, Chart5 for Windows analysis software was synchronized. Left ventricular end-systolic pressure (LVESP), left ventricular end-diastolic pressure (LVEDP), and the maximum rise and fall rate of left ventricular internal pressure (LV ± dp/dtmax) were recorded.

### CD31 Immunohistochemical Staining and Cardiac Capillary Density Measurement

The rats were sacrificed, the heart was removed, and the left ventricular myocardium was taken from the papillary muscles, fixed with 10% formaldehyde, and embedded in paraffin. The paraffin sections of myocardial tissue were stained with CD31 (Abcam, ab28364, USA) for immunohistochemistry and observed under an optical microscope. Eight slices were taken from each group, and 10 high-power fields (hpf) (400×) were randomly selected from each slice. The number of microvascular sections was calculated and averaged as the microvascular density (MVD) in the myocardial tissue.

### Transmission Electron Microscopy

A portion of the left ventricle was cut into 1-mm tissue blocks and fixed in 2.5% glutaraldehyde for more than 4 h for electron microscopy. Myocardial tissue was then immobilized with 1% osmium acid for 1 h and then rinsed and stained with 1% uranium acetate for 1 h. After dehydration with acetone and embedding with epoxy resin, myocardial tissue was cut into 1-µm sections and stained with toluidine blue. The ultrathin sections were cut from the block and studied under a JEM-1230 transmission electron microscope (JEOL of Japan).

### Statistical Analysis

Data analysis was performed using IBM SPSS Statistics 25.0 statistical software. The normal distribution of the measurement indicators was expressed as the mean ± SD. The multivariate sample means were compared for variance homogeneity test. The mean comparison between groups was analyzed by one-way ANOVA. The LSD-t test was used to compare the two groups. Dunnett’s T3 test was performed on the variance, and the difference was statistically significant at P < 0.05.

## Result

### Characterization of FGF1-nlip

**Figure 1:**
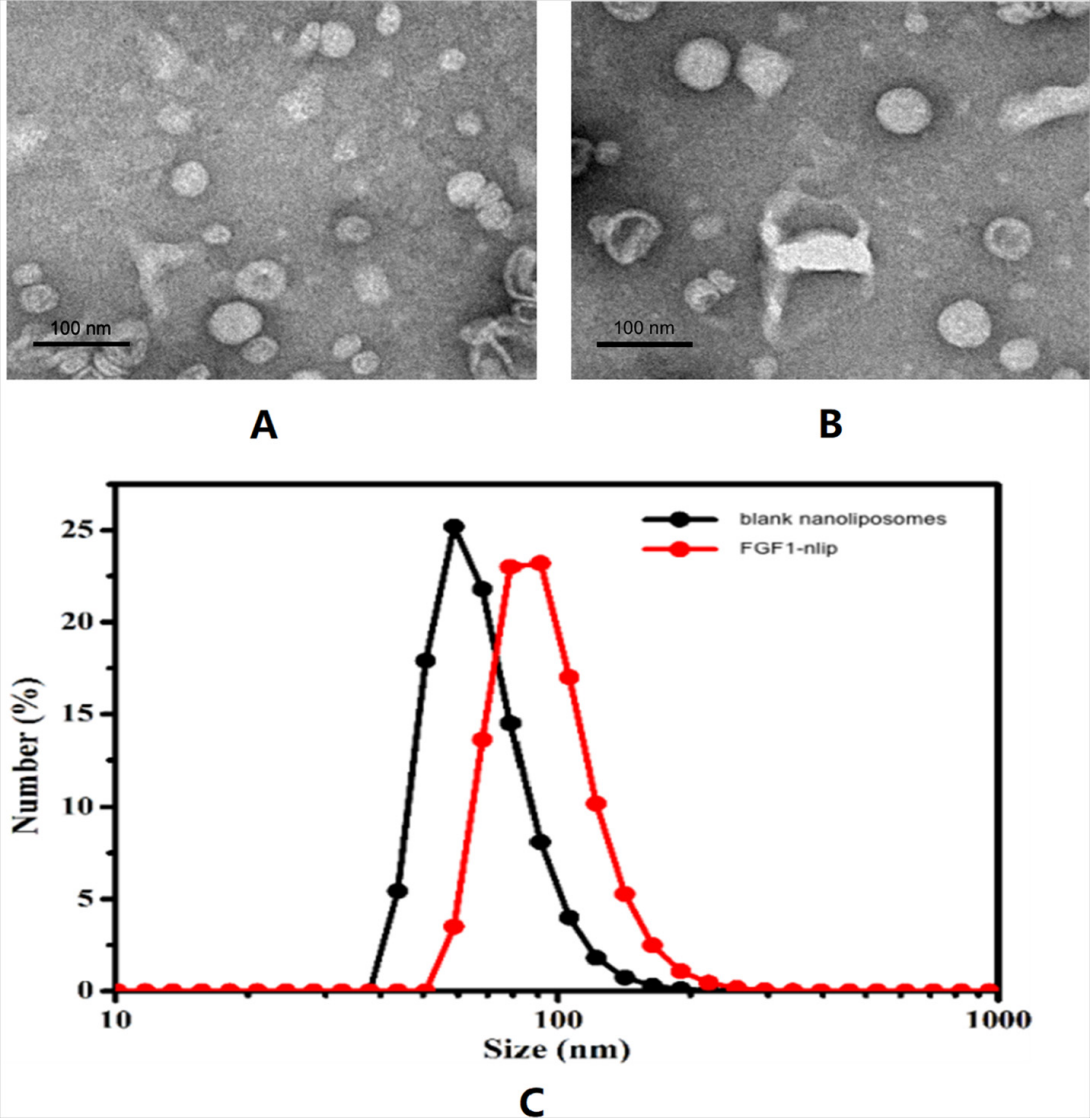
Transmission electron micrographs of blank nanoliposomes **(A)**, FGF1-nlip **(B)**, and the size distribution of blank nanoliposomes and FGF1-nlip **(C)**.

Observation of blank nanoliposomes and FGF1-nlip by transmission electron microscopy showed that the particle size distribution of the two was uniform, some adhesions existed, and the lipid bilayers were visible in the spherical shape (**Figure 1**). The average particle size of liposomes and FGF1-loaded liposomes was determined by the dynamic light scattering method to be 68.24 ± 1.61 nm and 96.23 ± 6.56 nm, respectively. The polydispersity index of the particle size was 0.092 ± 0.04 and 0.143 ± 0.02, respectively. Zeta potential measurements showed that the zeta potentials of the blank and drug-loaded liposomes were −1.885 ± 0.075 and −3.06 ± 0.01 mV, respectively. Further, the encapsulation efficiency measurement showed that the encapsulation efficiency of the FGF1 liposome was 73.52 ± 3.25%.

### General Condition of Experimental Rats

After 12 weeks of intervention, 4 rats in the DM model group and in the FGF1-nlip group died, 3 rats in the FGF1 solution group and the FGF1-nlip+UTMD group died, and no rats in the normal control group died. The number of rats in each group was 9 in the DM model group, 10 in the FGF1 solution group, 9 in the FGF1-nlip group, 10 in the FGF1-nlip+UTMD group, and 14 in the normal control group.

### Echocardiography Evaluation

Before intervention, there was no significant difference in LVEF and LVFS between the rats in each group (P > 0.05). After 12 weeks of intervention, the LVEF and LVFS in the DM model group were significantly lower than those in the normal control group (P < 0.05). Compared with the DM model group, the LVEF and LVFS of the FGF1 intervention group were significantly increased; compared with the other intervention groups, the rats in the FGF1-nlip+UTMD group were significantly increased (P < 0.05) (**Table 1**).

**Table 1:** Results of LVEF and LVFS (mean ± SD).

Group	n		LVEF(%)		LVFS(%)	
	Before intervention	After intervention	Before intervention	After intervention	Before intervention	After intervention
Normal control	14	14	82.87 ± 3.88	84.27 ± 2.80	46.19 ± 4.40	48.16 ± 3.18
DM model	13	9	83.51 ± 3.15	71.85 ± 2.78^a^	46.98 ± 3.72	36.21 ± 2.19^a^
FGF1 solution	13	10	83.87 ± 3.66	76.56 ± 3.95^b^_c_	47.31 ± 4.26	40.32 ± 3.41^b^_c_
FGF1-nlip	13	9	82.69 ± 4.24	77.09 ± 2.86^b^_c_	46.09 ± 4.87	40.80 ± 2.54^b^_c_
FGF1-nlip+UTMD	13	10	84.07 ± 3.41	80.87 ± 2.97^b^	47.51 ± 4.07	44.35 ± 3.05^b^

LVEF, left ventricular ejection fraction; LVFS, left ventricular fraction shortening. Data are Mean ± SD.

a *P* < 0.05 vs the normal control group.

b *P* < 0.05 vs DM model group.

c *P* < 0.05 vs FGF1-nlip+UTMD group.

### VVI Evaluation

**Figure 2:**
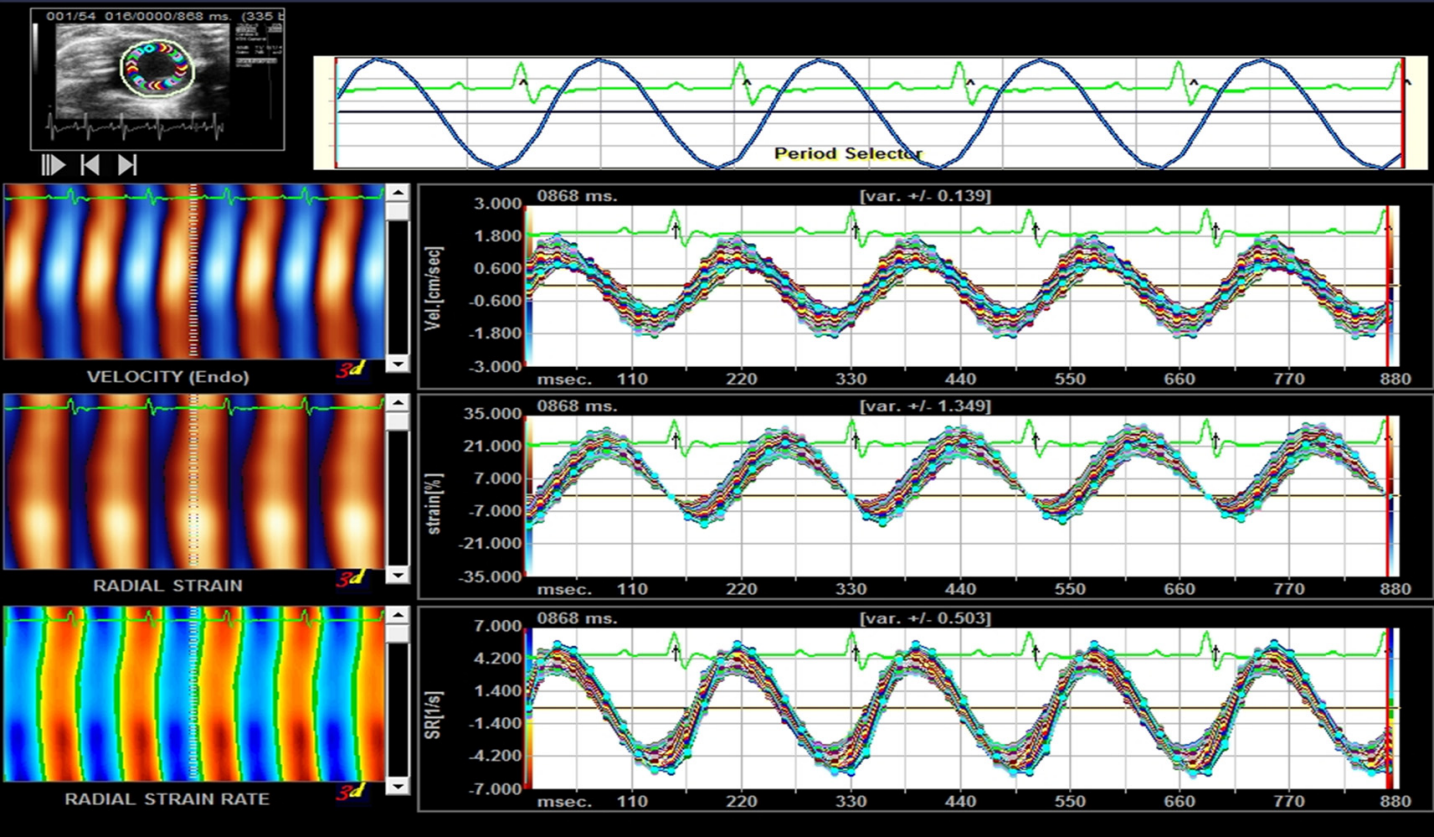
Representative picture of the myocardial velocity, radical strain, and radical strain rate curve provided by Sygno VVI.

As shown in **Figure 2**, the myocardial velocity, radial strain, and strain rate curves of 6 segments were obtained automatically by software. Before the intervention, there was no significant difference in Vs, Sr, and SRr among the groups (P > 0.05). After 12 weeks of intervention, Vs, Sr, and SRr in the DM model group were significantly lower than those in the normal control group (P < 0.05); Vs, Sr, and Sr in the FGF1 solution group, FGF1-nlip group, and FGF1-nlip+UTMD group were significantly higher than those in the model group (P < 0.05); those in the FGF1-nlip+UTMD group were higher than those in the other intervention groups (P < 0.05) (**Table 2**).

**Table 2:** Results of peak velocity, radical strain, and radical strain rate in the control and study groups (mean ± SD).

Group	n		Vs(cm/s)		Sr(%)		SRr(1/s)	
	Before intervention	After intervention	Before intervention	After intervention	Before intervention	After intervention	Before intervention	After intervention
Normal control	14	14	1.082 ± 0.081	1.106 ± 0.061	21.77 ± 1.72	22.43 ± 1.67	3.41 ± 0.24	3.37 ± 0.22
DM model	13	9	1.107 ± 0.063	0.751 ± 0.060^a^	21.49 ± 1.98	12.18 ± 1.32^a^	3.36 ± 0.27	1.73 ± 0.09^a^
FGF1 solution	13	10	1.111 ± 0.069	0.843 ± 0.052^b^_c_	22.08 ± 1.82	14.17 ± 1.01^b^_c_	3.31 ± 0.25	2.16 ± 0.19^b^_c_
FGF1-nlip	13	9	1.093 ± 0.058	0.858 ± 0.059^b^_c_	21.99 ± 1.71	14.66 ± 1.23^b^_c_	3.34 ± 0.29	2.23 ± 0.23^b^_c_
FGF1-nlip+UTMD	13	10	1.079 ± 0.082	0.968 ± 0.054^b^	22.55 ± 2.19	17.56 ± 1.06^b^	3.30 ± 0.24	2.80 ± 0.15^b^

Vs = systolic peak velocity; Sr = radial peak strain; SRr = radial peak strain rate.

a *P* ＜ 0.05 vs the normal control group.

b *P* ＜ 0.05 vs DM model group.

c *P* ＜ 0.05 vs FGF1-nlip+UTMD group.

### MCE Evaluation

**Figure 3:**
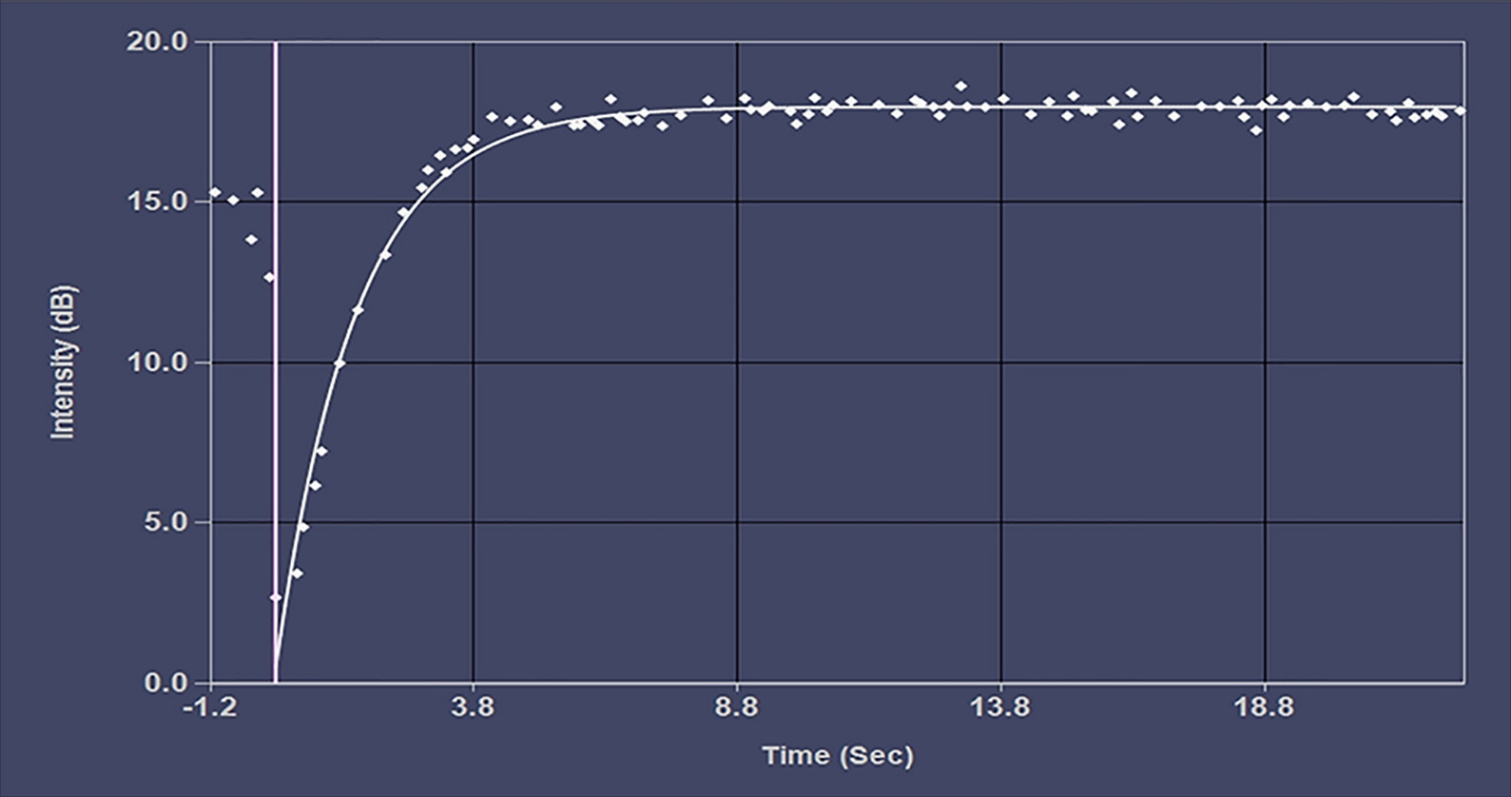
Representative picture of the time-intensity curve.

**Figure 3** shows the average acoustic intensity of the region of interest of each image frame. Before the experimental intervention, there was no significant difference in A, β, and A × β between the groups (P > 0.05). After 12 weeks of intervention, A, β, and A × β in the DM model group were significantly lower than those in the normal control group (P < 0.05). Compared with the DM model group, the FGF1 solution group and the FGF1-nlip group A and A × β were significantly increased (P < 0.05). Although there was an increasing trend of β, the difference was not statistically significant (P > 0.05), while the FGF1-nlip+UTMD group A, β, and A×β were significantly increased (P < 0.05); compared with the FGF1 solution group and FGF1-nlip group, the FGF1-nlip+UTMD group A and A × β were significantly increased (P < 0.05), and β increased slightly (P > 0.05) (**Table 3**).

**Table 3:** Results of A, β and A×β in the control and study groups (mean ± SD).

Group	n		A(dB)		β(s^-1^)		A×β(dB/s)	
	Before intervention	After intervention	Before intervention	After intervention	Before intervention	After intervention	Before intervention	After intervention
Normal control	14	14	25.24 ± 2.15	25.81 ± 2.80	0.81 ± 0.07	0.82 ± 0.07	20.42 ± 2.49	21.05 ± 3.16
DM model	13	9	24.96 ± 2.55	17.49 ± 1.68^a^	0.80 ± 0.05	0.71 ± 0.05^a^	20.07 ± 2.65	12.33 ± 1.73^a^
FGF1 solution	13	10	24.26 ± 2.37	19.81 ± 2.26^b^_c_	0.79 ± 0.05	0.74 ± 0.06	19.28 ± 2.27	14.63 ± 1.67^b^_c_
FGF1-nlip	13	9	25.52 ± 2.08	20.23 ± 1.87^b^_c_	0.81 ± 0.06	0.75 ± 0.05	20.60 ± 2.37	15.14 ± 2.07^b^_c_
FGF1-nlip+UTMD	13	10	24.67 ± 3.01	23.07 ± 2.31^b^	0.82 ± 0.07	0.78 ± 0.04^b^	20.35 ± 3.31	17.94 ± 1.53^b^

A = peak acoustic intensity; β = contrast agent perfusion rate; A×β = myocardial blood flow

a *P* ＜ 0.05 vs the normal control group.

b *P* ＜ 0.05 vs DM model group.

c *P* ＜ 0.05 vs FGF1-nlip+UTMD group.

### Hemodynamic Analysis

After 12 weeks of intervention, the LVESP, +dp/dtmax, and −dp/dtmax in the DM model group were significantly lower than those in the normal control group (P < 0.05), and LVEDP was significantly higher than that in the normal control group (P < 0.05). LVESP, +dp/dtmax, and −dp/dtmax in the FGF1 intervention groups were significantly higher than those in the DM model group (P < 0.05), and the levels were significantly lower than those in the model group (P < 0.05). The LVESP, +dp/dtmax, and −dp/dtmax in the FGF1-nlip+UTMD group were significantly higher than those in other FGF1 intervention groups (P < 0.05), and the LVEDP in the FGF1-nlip+UTMD group was significantly lower than that in the other FGF1 intervention groups (P < 0.05) (**Table 4**).

**Table 4:** The hemodynamic data in the *in vivo* experiment (mean ± SD).

Group	n	LVESP(mmHg)	LVEDP(mmHg)	+dp/dtmax(mmHg)	-dp/dtmax(mmHg)
Normal control	14	99.36 ± 7.46	3.17 ± 0.34	4926.79 ± 397.06	4438.64 ± 217.22
DM model	9	70.22 ± 5.29^a^	7.28 ± 0.51^a^	2967.33 ± 236.10^a^	2900.67 ± 151.77^a^
FGF1 solution	10	77.90 ± 5.08^b^_c_	6.03 ± 0.46^b^_c_	3682.90 ± 225.39^b^_c_	3373.90 ± 314.15^b^_c_
FGF1-nlip	9	79.67 ± 4.48^b^_c_	6.01 ± 0.67^b^_c_	3726.22 ± 251.26^b^_c_	3404.11 ± 226.63^b^_c_
FGF1-nlip+UTMD	10	88.70 ± 6.27^b^	4.83 ± 0.53^b^	4126.5 ± 340.45^b^	3835.30 ± 275.59^b^

Note : LVESP = left ventricular systolic pressure; LVEDP = left ventricular end diastolic pressure; ± dp/dt max = maximum rate of the rise and fall of left ventricular pressure.

a *P*＜ 0.05 vs the normal control group.

b *P*＜ 0.05 vs DM model group.

c *P*＜ 0.05 vs FGF1-nlip+UTMD group.

### Myocardial Capillary Density

**Figure 4:**
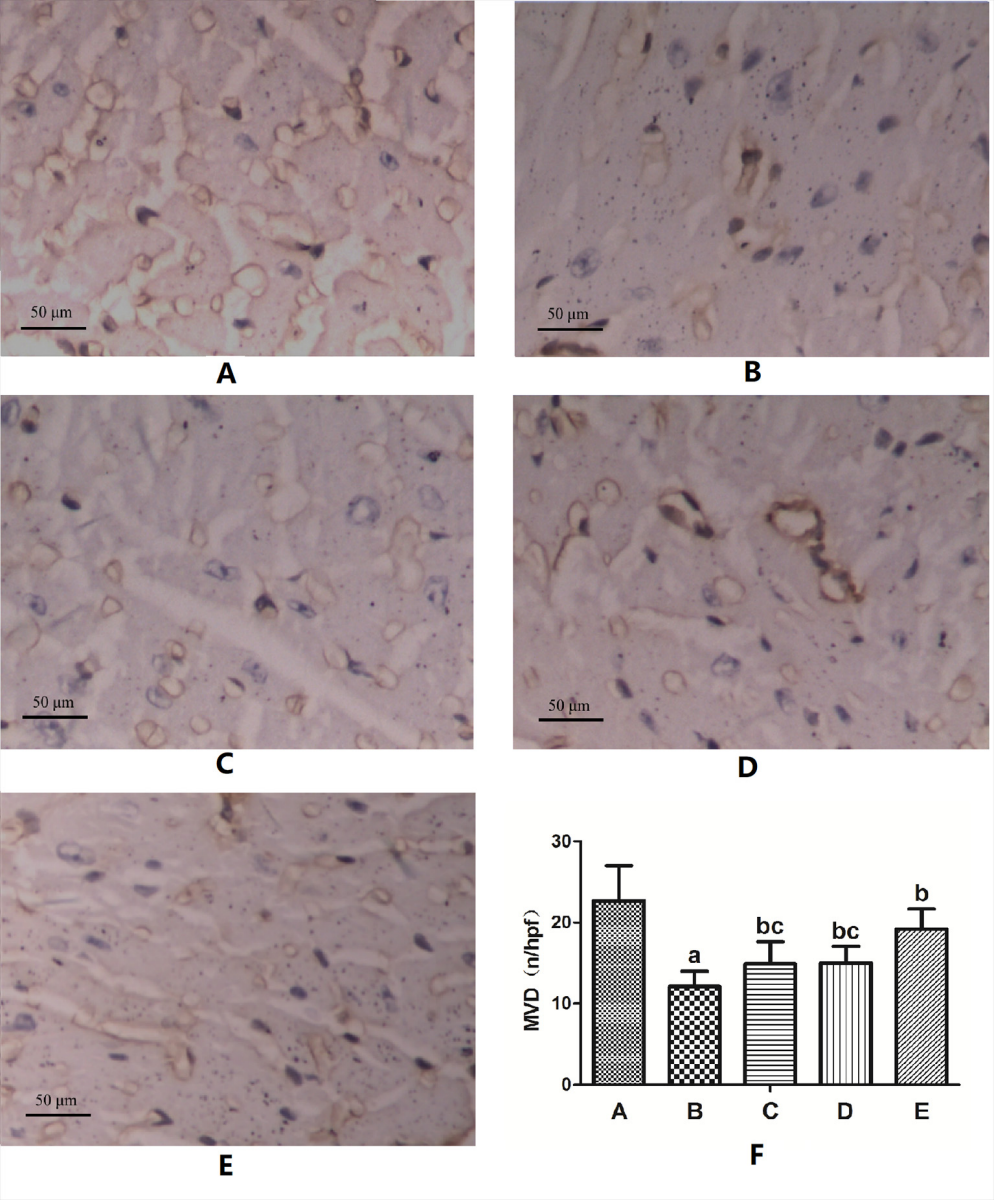
Representative images of CD31 immunohistochemical staining of MVD and the semi-quantitative analysis for all groups (400×) **(A)** Normal control group; **(B)** DM model group; **(C)** FGF1 solution group; **(D)** FGF1-nlip group; **(E)** FGF1-nlip+UTMD group; **(F)** Statistical histogram. ^a^P < 0.05 vs the normal control group, ^b^P < 0.05 vs the DM model group, ^c^P < 0.05 vs the FGF1-nlip+UTMD group).

CD31 immunohistochemical staining showed that the vascular endothelial cells were brown. The MVD of myocardial tissue in the DM model group was significantly lower than that in the normal control group (P < 0.05). The MVD in each FGF1 intervention group was significantly higher than that in the DM model group (P < 0.05). The MVD in the FGF1-nlip+UTMD group was significantly higher than that in the other FGF1 intervention groups (P < 0.05) (**Figure 4**).

### Electron Microscopic Findings

**Figure 5:**
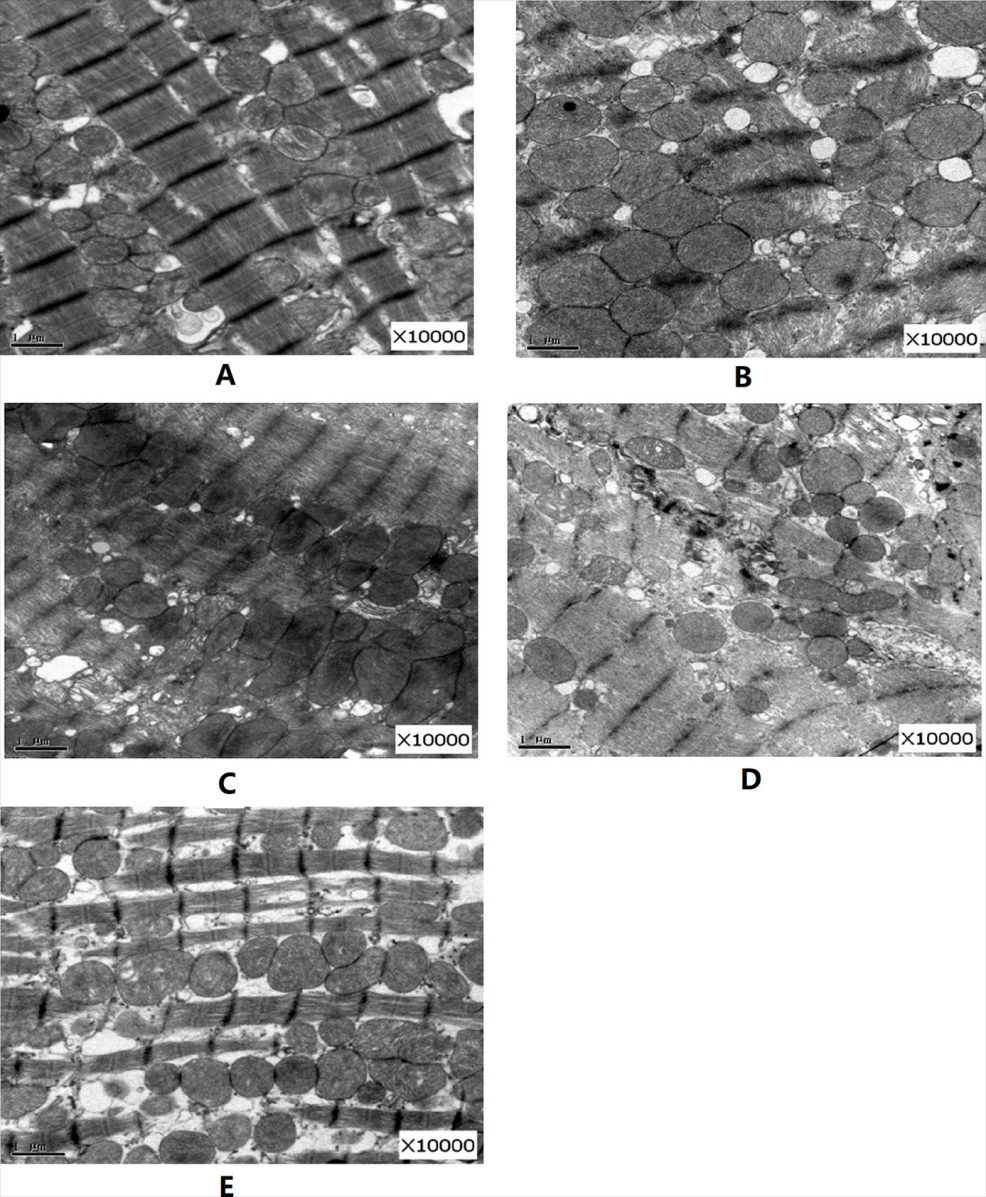
Representative pictures of electron micrographs (magniﬁcation 10000×) of left ventricular heart muscle sections from the rats of each group **(A)** normal control group; **(B)** DM model group; **(C)** FGF1 solution; **(D)** FGF1-nlip group; **(E)** FGF1-nlip+UTMD group)].

As shown in **Figure 5**, compared with the normal group, in the DM model group after 12 weeks, the Z line of cardiac muscle and myocutaneous fibers was arranged in a disordered manner, partly ruptured, with swollen aggregated mitochondria, and some vacuoles appeared. However, after 12 weeks of various forms of FGF1 prevention, the ultrastructure of the heart improved, especially in the fibrous filamentts and muscle fibers of the heart of rats in the FGF1-nlip+UTMD group, which were arranged neatly, and most of the mitochondria were normal. The results showed that the myocardial ultrastructure of DM rats was improved after different forms of FGF1 prevention, but compared with other forms of prevention, FGF1-nlip combined with UTMD technology had the most obvious preventive effect.

## Discussion

In this study, the rat model of DM was established by intraperitoneal injection of STZ. Routine echocardiography, the VVI technique, and RT-MCE were performed before drug intervention. No significant difference was found in cardiac function and the blood perfusion index among each group. After 12 weeks of feeding, DM rats underwent routine echocardiography, VVI, and cardiac catheterization techniques. The results showed that the LVEF, LVFS, and VVI indexes (Vs, Sr, and SRr), LVESP, and ± dp/dtmax in the DM model group were significantly lower than those in the normal control group (P < 0.05), while LVESP in the DM model group was higher than those in the normal control group (P < 0.05). RT-MCE showed that A, β, and A × β in the DM model group were significantly lower than those in the control group. This indicated that obvious abnormalities in left ventricular systolic function and blood flow perfusion appeared in DM rats at the end of 12 weeks. Histological observation also confirmed that the MVD value of myocardial tissue in DM rats was significantly lower than that in the control group, and there were pathological changes such as disordered filament arrangement, mitochondrial swelling and degeneration. Therefore, it is very important to prevent the occurrence of DCM by intervening before the obvious pathological changes in myocardium in DM rats.

FGF1 is a type of cell growth factor with many biological activities. It has a high affinity for heparin. FGF1 can promote the proliferation of vascular endothelial cells and smooth muscle cells, thereby promoting angiogenesis, alleviating myocardial ischemia, and improving cardiac function (Cochain et al., 2013; Zhang et al., 2016). In previous studies, in various animal models of myocardial ischemia or infarction, FGF1 increased the regional myocardial blood flow and the density of capillaries and arterioles and improved ventricular function (Zhao et al., 2012). Other studies have shown that non-mitotic FGF1 can prevent DCM (Zhang et al., 2013) by inhibiting oxidative stress and injury. Therefore, FGF1 is a potentially valuable therapeutic agent for the prevention and treatment of DCM.

In order to increase the stability of the drug, the reverse phase evaporation method (Yue et al., 2014; Mehrabi et al., 2016) was used to prepare nanoliposomes encapsulated with FGF1. Through the analysis of its characteristics, it was confirmed that the drug-loaded lipids had a round appearance, small particle size (<100 nm), good dispersion (polydispersity index < 0.15), and a certain Zeta potential, which could maintain good stability (**Figure 1**). Compared with traditional liposome preparation technology, the method adopted in this study has a shorter preparation time, and the average temperature of the whole preparation process is controlled below 25°C, which can maintain the biological activity of protein drugs to a great extent. This shows that the preparation method can efficiently achieve the inclusion of FGF1 and is suitable for *in vivo* application. In order to increase the local concentration of FGF1 in the heart, exert the therapeutic effects to a greater extent, and reduce the influence on the whole body (e.g., liver, spleen, and kidney), this study injected FGF1-nlip mixed with SonoVue microbubbles into DM rats *via* the caudal vein. After myocardial development, UTMD was used to explode microbubbles to increase the dose of FGF1-nlip released into the myocardium and exert its corresponding biological effects.

After 12 weeks of intervention, routine echocardiography and VVI (**Tables 1**–**2**) showed that LVEF, LVFS, and VVI (Vs, Sr, and SRr) in the FGF1 intervention group were significantly higher than those in the DM model group (P < 0.05), while LVEF, LVFS, and VVI (Vs, Sr, and SRr) in the FGF1-nlip+UTMD group were significantly higher than those in the other FGF1 intervention groups (P < 0.05), which was confirmed by hemodynamic studies (**Table 4**). The results showed that the FGF1-nlip+UTMD group had the least damage to left ventricular systolic function in DM rats. In addition, the ultrastructural changes of the FGF1-nlip+UTMD group were the least. Therefore, this method can be used as an effective strategy to prevent the deterioration of cardiac function in DM rats.

The results of RT-MCE in this study (**Table 3**) showed that after 12 weeks of intervention, the levels of A and A × β in the FGF1 solution group and FGF1-nlip group were significantly higher than those in the DM model group (P < 0.05), while the levels of A, β, and A × β in the FGF1-nlip+UTMD group were significantly higher than those in the DM model group (P < 0.05), and A and A × β were significantly higher than those in the other FGF1 intervention groups. To improve myocardial microcirculation, the application of FGF1-nlip combined with UTMD has the best preventive effect. This was confirmed by CD31 immunohistochemical assay (**Figure 4**).

In addition, there was no significant difference in left ventricular function and blood perfusion between the FGF1 solution group and the FGF1-nlip group. The reason is that the drug encapsulated by liposomes alone lacks targeting ability and is easily captured by the reticuloendothelial system *in vivo* (Ishihara et al., 2012; Hsu et al., 2014). It is difficult to maximize the efficacy of the drug in cardiac aggregation. Combined with UTMD, the cavitation effect and mechanical action produced by the instantaneous blasting of ultrasound microbubbles can cause temporary open pores (Chen et al., 2013; Qian et al., 2018; Zhao et al., 2018) on the endothelial cell membrane and capillaries of the myocardium. The target delivery of FGF1-nlip to the myocardium can realize the rapid release of the target and rapid penetration, thus realizing the myocardial transmission of FGF1 and exerting its biological function.

In the present study, although each intervention group had a significant improvement in cardiac function, the survival rate was lower than that in the normal control group. The cause of death in rats is related to the deterioration of the physical condition of rats caused by diabetes injury, and may be related to the influence of anesthetic drugs. Because the animals could not cooperate effectively, the rats in the intervention group had higher requirements for anesthesia depth to ensure the stability and effectiveness of the intervention. The normal control group did not undergo relevant intervention, so there was no death.

In conclusion, the application of FGF1-nlip combined with UTMD technology can efficiently deliver FGF1 to the heart of DM rats and prevent abnormal left ventricular systolic function and myocardial perfusion in DM rats. The changes in left ventricular systolic function and blood perfusion in DM rats can be detected by VVI and RT-MCE technology, which provides a more effective basis for the evaluation of DCM therapeutic effects.

The limitations of this study are as follows: First, UTMD technology is used to promote the entry of FGF1-nlip into cells, thus causing a certain degree of damage to cells. It is necessary to further optimize the parameters of ultrasound irradiation for local release of the highest drug concentration in the case of minimal tissue damage. Second, because the pathogenesis of DCM is complex and the effect of FGF1 alone is limited, whether it is necessary to combine other drugs to improve the efficacy needs further study.

## Data Availability Statement

All datasets generated for this study are included in the article/supplementary material.

## Ethics Statement

The animal study was reviewed and approved by the Animal Ethics Committee of Wenzhou Medical University.

## Author Contributions

LZ: Overall experimental design, data analysis, paper writing. C-LS: Acquisition and analysis of ultrasound Image of animals. J-ML: Histopathological detection and analysis, wzyxyljmin@163.com. Y-LM: Feeding of experimental animals, imayulei@163.com. NY: Pharmaceutical intervention in laboratory animals. X-QT: Research overall guidance, paper revision, funding support. Y-ZZ: Preparation of nanoliposomes, revision of papers, financial support.

## Funding

This research was supported by the Chinese National Natural Science Funds (Grant No. 81571696, U1704175), Medicine Grant from Wenzhou Bureau of Science and Technology (Y20170049), Key Research and Development Program of Zhejiang province (Grant No. 2018C03013).

## Conflict of Interest

The authors declare that the research was conducted in the absence of any commercial or financial relationships that could be construed as a potential conflict of interest.
